# Integrated approach to the understanding of the degradation of an urban river: local perceptions, environmental parameters and geoprocessing

**DOI:** 10.1186/s13002-015-0054-y

**Published:** 2015-09-15

**Authors:** Carolina A. Collier, Miguel S. de Almeida Neto, Gabriela MA Aretakis, Rangel E. Santos, Tiago H. de Oliveira, José S. Mourão, William Severi, Ana CA El-Deir

**Affiliations:** Post-Graduation Program of Ethnobiology and Conservation of Nature, Department of Biology, Federal Rural University of Pernambuco, P.O. Box 52171–900, Recife, Brazil; Post-Graduation Program of Development and Environment, Centre of Philosophy and Human Sciences, Federal University of Pernambuco, P.O. Box 50740–530, Recife, Brazil; Post-Graduation in Ecology, Department of Biology, Federal Rural University of Pernambuco, P.O. Box 52171–900, Recife, Brazil; Department of Biology, State University of Paraíba, P.O. Box 58000–000, Campina Grande, Brazil; Department of Fisheries and Aquaculture, Federal Rural University of Pernambuco, P.O. Box 52171–900, Recife, Brazil; Department of Biology, Federal Rural University of Pernambuco, P.O. Box 52171–900, Recife, Brazil

**Keywords:** Geographic information system, Ethnobiology, Ecosystem services, Environmental quality, Freshwater fish, Eutrophization

## Abstract

**Background:**

The use of interdisciplinary approaches such as the proposed report provides a broad understanding of the relationship between people and the environment, revealing reliable aspects not previously considered in the study of this relationship. This study compiled evidence on the environmental degradation of an urbanized river over the past few decades, providing a diagnosis of the consequences of this process for the river, its ichthyofauna, and the local human population.

**Methods:**

The study was focused on the Beira Rio community on the Capibaribe River in the municipality of São Lourenço da Mata, Pernambuco, Brazil. Data were collected using geoprocessing and ethnobiological approaches, as well as environmental parameters. This research was conducted with the most experienced long-term residents in the local community, through interviews and participatory methodologies to recovering information about the river environment, its ichthyofauna and its environmental services for the last decades.

**Results:**

According to the GIS analysis, the study area was subject to an accelerated process of urbanization, with the total urban area increasing from 73 565, 98 m^2^ in 1974 to 383 363, 6 m^2^ in 2005. The informants perceived the urban growth, especially in the late twentieth century, being this period recognized as the phase of greatest negative changes in the river environment. The perceived decline of fish stocks was indicated by the community as one of the effects of river degradation. According to the interviews, the deterioration of the river affected the ecosystem services and the relationship of the adjacent human community with this ecosystem. The environmental data indicated that the river is suffering eutrophization and has fecal coliform concentrations 160 times higher than the maximum level permitted by Brazilian legislation.

**Conclusions:**

The interdisciplinary approach used in this research allowed the understanding of the degradation process of an urban river and some negative effects through the integration of environmental data, GIS and the local knowledge, revealing the complementarity of obtained data and the effectiveness of implementation of this approach.

## Resumo

**Introdução:**

A utilização de abordagens interdisciplinares, como nesta pesquisa, provê uma ampla compreensão da relação entre as pessoas e o ambiente, revelando aspectos relevantes não considerados anteriormente no estudo desta relação. Este estudo compilou evidências sobre a degradação ambiental de um rio urbano ao longo das últimas décadas, provendo um diagnóstico das consequências deste processo sobre o rio, sua ictiofauna e a população humana local.

**Métodos:**

Este estudo foi realizado na comunidade Beira Rio no rio Capibaribe, município de São Lourenço da Mata, Pernambuco, Brasil. Os dados foram coletados utilizando geoprocessamento e abordagens etnobiológicas, bem como análises abióticas. Esta pesquisa foi conduzida com os moradores mais antigos da comunidade local, através de entrevistas e metodologias participativas para recuperar informações sobre o ambiente do rio, sua ictiofauna e seus serviços ambientais nas últimas décadas.

**Resultados:**

De acordo com as análises de SIG, a área de estudo sofreu um acelerado processo de urbanização, com a área urbana total aumentando de 73 565,98 m^2^, em 1974, para 383 363,6 m^2^ em 2005. Os informantes perceberam o crescimento urbano, principalmente no final do século XX, sendo a transição deste século reconhecida como o período de maiores modificações negativas no ambiente do rio. O declínio percebido dos estoques pesqueiros foi apontado pela comunidade como um dos efeitos da degradação do rio. De acordo com as entrevistas, a deterioração do rio comprometeu os serviços ambientais e a relação da comunidade humana adjacente com este ecossistema. Os dados ambientais indicaram que o rio está sofrendo eutrofização, e apresenta uma concentração de coliforme fecal 160 vezes maior que o máximo permitido pela legislação brasileira.

**Conclusões:**

A abordagem interdisciplinar utilizada neste estudo permitiu compreender o processo de degradação de um rio urbano e alguns de seus efeitos negativos através da integração de dados ambientais, SIG e do acesso ao conhecimento local, demonstrando a complementariedade dos dados obtidos e a efetividade deste modelo de abordagem.

## Background

Throughout history, human beings have tended to agglomerate along the margins of watercourses, where they have access to important resources, such as water, food, and raw materials [1]. These areas tend to be naturally more fertile, favoring agriculture and ultimately contributing to their anthropogenization [1]. In the present day, hydrological resources are exploited in a number of different ways, ranging from domestic, agricultural, and industrial consumption, through the removal and dilution of effluents and solid waste, to the generation of electricity [2].

The initial consequence of the human occupation of river margins is deforestation, due to the demands for physical space [3]. This results in soil exposure, leading to erosion, excessive leaching of nutrients, and the eventual siltation [3]. The development of urban environments leads to an increase of nutrients concentration and fecal coliforms due to the discharge of domestic effluents [4]. This contamination promotes modifications in the physical-chemical characteristics of the water, and in turn, the quality of the aquatic environment [5]. The addition of nutrients promotes ecosystem-level problems, such as algal blooms, biochemical oxygen demand increase and hypoxia [6]. Fecal contamination also contributes to hypoxia, as well as human illnesses [6].

The contamination of water bodies impedes the exploitation of their resources by the population, in particular by reducing supplies for domestic consumption and usage [7]. The degradation of aquatic ecosystems may also result in a decline in fishery stocks [8], which may affect the economic income of some riverine communities. In addition to provide water and economic resources, aquatic ecosystems providing other ecosystem services that are less recognized by the society, such as cultural identity, spiritual and religious values, recreational activities, among others, that contribute to quality of life of human populations [9]. Local people know, perceive and use the resources along their history with the environment where they live, therefore they are also responsible for the environmental changes occurring around them [10] This relationship leads to a knowledge accumulation about the environmental changes and processes [11]. Accessing perceptions of the local population makes it possible to identify these changes and their causes [12, 13]. Therefore, Hanazaki et al. [14] highlight the importance of including the perception of local human populations in obtaining information to help clarifying questions about the environmental changes occurring. These environmental changes and their relationship with the human societies have been better understood through approaches of historical ecology, which seeks to understand the conformation of contemporary and past cultures and landscapes [15].

The understanding of the relationship between man and nature, as well as the anthropogenization of the environment, has been advancing in recent years through the adoption of interdisciplinary approaches, which combine different methods and theoretical perspectives to provide integrated insights into these phenomena (e. g. [16, 17]). In this context, the present study integrated data on environmental data related to fecal coliform and trophic state index (TSI), Geographic Information System (GIS) analyses, and local knowledge provided by the resident population, with the objective of understanding the degradation of an urban river in northeastern Brazil over the past three decades, and in particular the consequences of this process for the river, its ichthyofauna, and the local riverside community.

## Methods

### Study area

The basin of the Capibaribe River is located in the Brazilian state of Pernambuco, where it flows through 42 different municipalities within a total area of 7454 km^2^ [18]. The Capibaribe runs 280 km from its headwaters in the municipality of Poção to the Atlantic Ocean in the state capital, Recife [18]. The lower Capibaribe flows through the Metropolitan Region of Recife (MRR), the sixth largest urban center in Brazil [19].

**Fig. 1:**
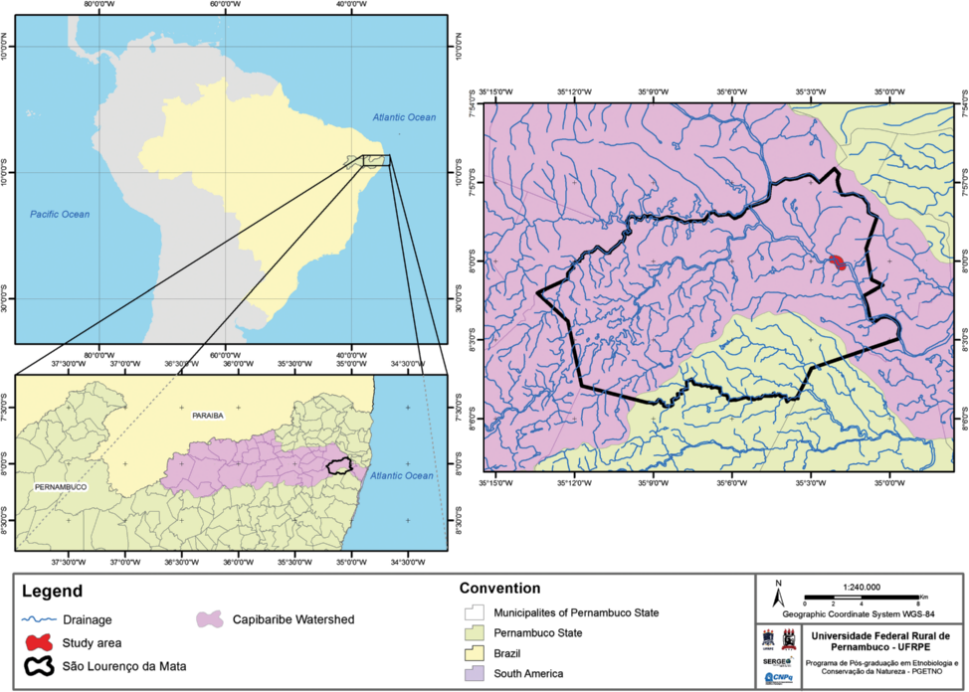
Study area, lower Capibaribe river, municipality of São Lourenço da Mata, Pernambuco, Brazil. Location of the study area on the lower Capibaribe River in the municipality of São Lourenço da Mata, Pernambuco, Brazil

São Lourenço da Mata is one of the municipalities that make up the MRR. In 2014, it had an estimated population of 109,298 inhabitants, of which 94 % reside in the urban zone [20]. The community known as Beira Rio is located in this municipality between the coordinates 7°59’57.414” S, 35°2’18.434” W and 8°00’10.434” S, 35°1’3.929” W (Fig. 1). Beira Rio was founded in the 1980s, and subsequently developed into an urban center characterized by a lack of planning and precarious infrastructure, whose streets being paved only at the end of the 1990s. Domestic effluents are discharged directly into the river to this day. These peripheral and unplanned growth together with the lack of basic infrastructure are a common situation on most Brazilian cities [21].

### Land use and land cover

Four maps of land use and land cover were produced in order to characterize the process of urban expansion since the establishment of the Beira Rio community. For this, a buffer of 250 m was established around the stretch of the river selected for evaluation in the present study (Fig. 1). Three maps were then produced based on the interpretation of Panchromatic B & W vertical aerial photographs obtained from the Pernambuco State Planning and Research Agency (Condepe/Fidem). Two photographs were available for 1974, one for 1981, and two for 1997. Given the lack of more recent aerial photographs, the fourth map was produced based on a Google Earth image for 2005.

The images were georeferenced using the Universal Transversal Mercator Projection System and the WGS-84 datum. The data were then vectored according to land use and land cover, being classified as (i) vegetation, (ii) urban area, (iii) river, and (iv) roads. A 1:15 000 scale was adopted following vectoring. The maps were produced in ArcGIS 9.3, licensed to the Observatory of the Geographical Sciences Department at the Federal University of Pernambuco (UFPE) in Recife, Brazil.

### Environmental parameters

To compile a history of the water quality of the study area, data were obtained from the CB-72 monitoring station (7° 59’57.672” S, 35° 2’ 0.406” W) situated inside the delimited area to community and realized by the Pernambuco State Environment Agency (CPRH). The CPRH provided Trophic State Indices (TSI) and fecal coliform concentrations for the 23 year period between 1991 and 2013. As data were lacking for some months of the year, only dry season values were used for analysis.

The CPRH considers 160 000 MPN/100 ml as the maximum value for fecal coliform concentrations. The values recorded were compared with the limits for human use established by the Brazilian National Council for the Environment (CONAMA) through Resolution 375/05 for rivers contained in class 2 [22]. The CPRH used total phosphorus values to calculate the TSI, classified in six categories (Table 1).

**Table 1:** Categories used for the classification of the Trophic State Index

Category	Trophic State Index (TSI)
Ultraoligotrophic	TSI ≤ 47
Oligotrophic	47 < TSI ≤ 52
Mesotrophic	52 < TSI ≤ 59
Eutrophic	59 < TSI ≤ 63
Supereutrophic	63 < TSI ≤ 67
Hypereutrophic	TSI > 67

Categories used for the classification of the Trophic State Index. Source: CETESB (2013)

### Local knowledge

Local informants were selected using the snowball approach [23], in order to identify the most experienced long-term residents of the Beira Rio community. These residents were considered to be those individuals who had lived continuously in the community for at least two decades. The objectives of the study were explained to all informants, who were invited to sign a free and informed consent form, as required by federal resolution CNS 466/12 [24]. This study was approved by the Committee for Ethics in Research Involving Human Subjects at the University of Pernambuco (UPE) under process number 38385814 8 0000 5207.

Semi-structured interviews were used to obtain information from 18 local residents, focusing on their relationship with the river in the past and the present day, regarding fishing activities, conservation of the river and its ichthyofauna, and the causes and consequences of the environmental modifications of the river. The information provided on the modifications of the river, and the causes of these modifications, was grouped into categories. The information provided in the interviews was also analyzed using the collective subject technique of Lefevre [25], in which the key expressions are identified and used to collate a “collective discourse” of the principal ideas expressed by the informants.

A free list technique [26] was used to compile an inventory of the fish species known to the population. A checklist [27] was used to confirm the names cited and identify the species based on photographs of specimens taken *in situ*. To understand the representation of the informants with regard to the historical modifications of the river, a participative Timeline approach was used, in which the subjects were asked to remember historic events related to the modification of the river [24]. Although 18 informants were invited to participate in this process, only six (33,3 %) actually did.

The participative Historical Graph method [28] was used to obtain information on the abundance of fish in the river. In this participatory approach it was requested of the informants to indicate the abundance of confirmed species in checklist using integer values between 0 and 10. In this case, all the 16 informants who practice or have practiced fishing on the river were invited to participate and provide estimates of the abundance of the different fish species in the Capibaribe during the period in which they fished the river. Four informants provided information on the abundance of fish in the river, including the only fisherman who continues to work in the river in Beira Rio community so far.

## Results

### Urban expansion

**Fig. 2:**
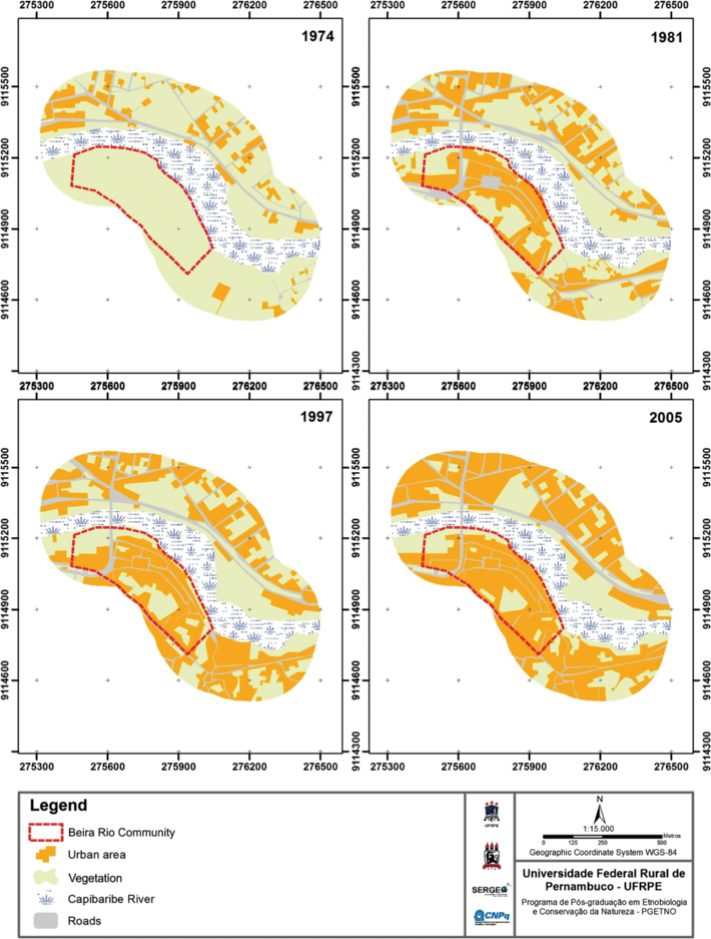
Land use and land coverdra, lower Capibaribe River, municipality of São Lourenço da Mata, Pernambuco, Brazil. Land use and land cover of a stretch of the lower Capibaribe River in São Lourenço da Mata, Pernambuco (Brazil), in 1974, 1981, 1997, and 2005

Between 1974 and the inception of the Beira Rio community, in the 1980s, the urban area surrounding the river increased from 73 565 98 m^2^ in 1974 to 240 555 12 m^2^ in 1981 (Fig. 2). This process resulted in the reduction of the area of vegetation from 507 944 62 m^2^ to 321 196 38 m^2^ by 1981 (Fig. 2). After the community establishment, the urban area increased to 297 489 80 m^2^ between 1981 and 1997, representing an expansion of 24 % over this period (Fig. 2). By 2005, the urban area had grown to 383 363 60 m^2^, indicating an increase of 29 % between 1997 and 2005 (Fig. 2).

### Environmental parameters

**Fig. 3:**
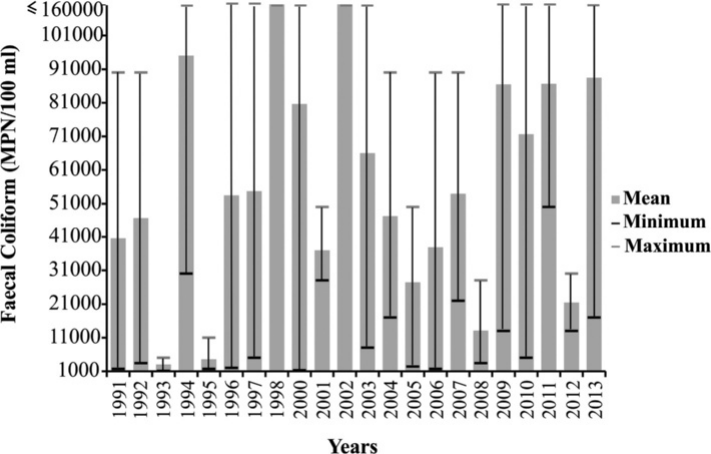
Faecal coliform concentrations (MPN/100 ml), lower Capibaribe River in São Lourenço da Mata, Pernambuco, Brazil. Source: CPRH. Faecal coliform concentrations (MPN/100 ml) recorded between 1991 and 2013 at monitoring station CB-72 on the lower Capibaribe River in São Lourenço da Mata, Pernambuco, Brazil. Source: CPRH

Fecal coliform concentrations varied between 1100 and ≥160 000 MPN/100 ml (Fig. 3). As the lowest of these values exceeds the upper limit of 1000 MPN/100 ml defined by CONAMA 357/05, the river water would have been unfit for human activities throughout the monitoring period (1991–2013).

**Fig. 4:**
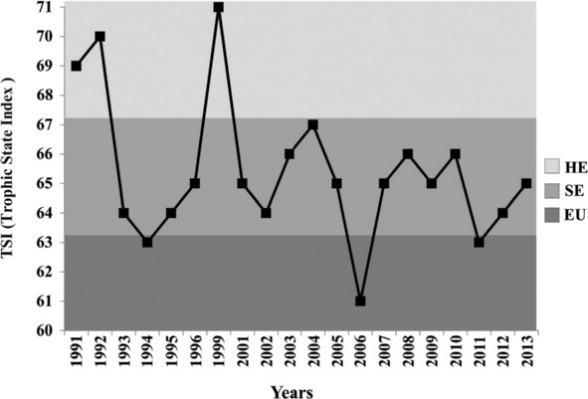
Trophic State Indices (TSI), lower Capibaribe River in São Lourenço da Mata, Pernambuco, Brazil. Source: CPRH. Trophic State Indices (TSI) recorded between 1991 and 2013 at monitoring station CB-72 on the lower Capibaribe River in São Lourenço da Mata, Pernambuco, Brazil. Source: CPRH. EU = eutrophic, SE = supereutrophic, HE = hypereutrophic

Trophic State Index (TSI) varied from 61 to 71 during the period, corresponding to a classification of eutrophic to hypereutrophic (Fig. 4). Most of the values were between 63 and 67, indicating that the river was supereutrophic during most of the monitoring period. The river has thus been characterized by high levels of eutrophization since 1991, reaching a peak in 1999 (Fig. 4).

### Local knowledge on changes in the river and the fish fauna

**Fig. 5:**
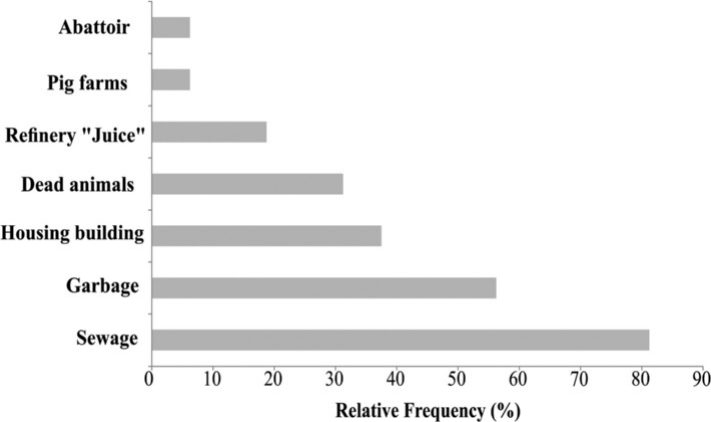
Perceived causes of degradation of lower Capibaribe River, according to informants interviewed in the Beira Rio community. Relative frequency of the perceived causes of the degradation of the lower Capibaribe River, as reported according to informants interviewed in the Beira Rio community, São Lourenço da Mata, Pernambuco, Brazil

The degradation of the river was acknowledged by 100 % of the interviewees, who indicated negative modifications in the river and its fish fauna. The principal modifications reported referred to alteration of the color (81 %) and odor (19 %) of the water, although one (6 %) also mentioned changes in water levels. Most (88 %) of the informants reported a long-term decline in the abundance of fish. The causes of these modifications were identified (Fig. 5) as primarily the discharge of sewage (81 % of the informants), garbage (56 %), and dead animals (31 %) directly into the river, in addition to the construction of houses (38 %).

All (100 %) of the informants reported consuming fish from the river in the past, but only two (11 %) still eat fish caught in the river. One of the consequences of the pollution of the river reported by the informants was the abandoning of fishery activities. Most (89 %) of the informants reported having fished on the river in the past, but 94 % of these individuals confirmed they no longer do this, and only one fisherman is still active.

According to the collective discourse extracted from the information obtained in the interviews (Table 2), no loss of quality was observed in the river during the 1980s, when the water was still transparent, and the bottom was sandy. During this decade, the river was a source of drinking water and was used for other domestic activities, and many local fishermen exploited the abundance and diversity of fish in the river to support their families. At the beginning of the 1990s, the characteristics and environmental services of the previous decade were maintained, although subsequent population growth, combined with the extraction of materials from the river for the construction of houses, led to the beginning of the worsening of the river. At the turn of the century, the informants report an increase in population growth and the degradation of the quality of the river, which was now considered to be “very polluted”, with “black water”, and a “bad smell of faeces”. During this decade, the river sediment changed from sandy to muddy, and the river ceased to represent a source of services and resources for the local population, due to the fact that the water had become unfit, and the few surviving fish are contaminated, and in fact, the mere contact with the water may have health risks, due to the presence of “schistosomiasis and germs” (Table 2).

**Table 2:** Collective discourse on the process of degradation of the lower Capibaribe River

Decade	Collective subject discourse
1980-1989	“In the old days, there was just bush and a lake, there were no houses on the bank of the river. The river was clean, with transparent water, so you could see the sandy bottom. The water was drinkable, and the people bathed, and washed clothes and dishes. There was a lot of fish, of many different types. The river supported the local families, providing them with fish. Everybody ate a lot of fish from the river.”
1990-1999	“There was just bush and mud shacks. There weren’t so many houses. The river was clean, with transparent water, it was possible to see the bottom and the fish. We used to bathe, and wash dishes and clothes. There was a lot of fish, a lot of people fishing. The river supported the local families, providing them with fish. The community began to fill up with houses, the people took sand and stones from the river to build their houses, and the river began to get worse.”
2000-present day	“The Beira Rio Community population has increased a lot, there are many houses. The river has changed completely, it is very polluted, it is really gross. The river is dirty now, it is always muddy, with black water. Nowadays it stinks, smells like feces with mud on the bottom. The river is full of garbage, it’s only good for a sewer. Nobody can go in to the river, if somebody steps in the water, they get sick. The river is full of schistosomiasis and germs. The fish are all gone, there’s almost no fish now, they have disappeared, it’s a disgrace, there’s nothing left! You never see anyone fishing now. The fish come up covered in sludge, they are black, like the river. In the old days, everyone ate fish, but now nobody does. We are pish and afraid to eat fish because the river is so polluted.”

Collective discourse on the process of degradation of the lower Capibaribe River, constructed from the reports of informants interviewed in the Beira Rio community, São Lourenço da Mata, Pernambuco, Brazil

According to the Timeline, the community was established by squatting on land previously used for planting sugarcane, and was based on subsistence agriculture and fishing (Table 3). At the beginning of the 1980s, there were few houses, and the first major growth in the local population began in 1987. During this first decade, the discharge of a “juice” from the Tiúma sugar refinery was a recurring event (Table 3). This typically resulted in unhealthy odors and fish mortality over a period of approximately one week. Despite the clear noxious effects of this phenomenon on the river and its ichthyofauna, local residents would take advantage of these events to capture fish, which were typically found floating on or near the surface, in an intoxicated condition (Table 3).

**Table 3:** Timeline of the historical events leading to the modification of the lower Capibaribe River

Decade	Event or activity	Consequences	Description	Use
1980-1989	Community established (1980)	Occupation of land once used as sugarcane plantations	Clean river	Bathing
	Plantations of okra and cassava	Uninformed		Washing clothes and dishes
	Discharge of “juice” from the Tiúma sugar refinery	Bad-smelling water, fish mortality, and capture of intoxicated fish.	Many fish	Drinking water
	Increase in the number of houses (1987)	Initial population growth		Fishing
1990-1999	Tiúma refinery closes (1994)	Discharge of “juice” ends	Clean river (until 1998)	Uninformed
	Fish stocking in adjacent areas (1994)	Appearance of carpa, tambaqui, and cará-trovão	Many fish (until 1998)	
	Flood (1994)	Destruction of houses		
	Streets paved (1998)	Uninformed	River begins to “get much worse” (1998/1999)	
	Major growth in the size of the community (1998/1999)	Increase in the discharge of sewage, garbage and dead animals into the river		
2000-present day	Garbage truck collection begins on a regular basis (2000)	Reduction in the discharge of solid refuse into the river	River polluted since 2000	Uninformed
	Houses built (2000)	Increase in the discharge of sewage into the river		
	Dicharge of a “juice” of unknown origin (2002)	Uninformed		
	Appearance of the beta, known as the “sewage fish” (2004)	Uninformed		
	Flood (2011)	Destruction of houses		

Timeline of the historical events leading to the modification of the lower Capibaribe River, produced from the information supplied by informants interviewed in the Beira Rio community, São Lourenço da Mata, Pernambuco, Brazil

During the 1990s, carpa, tambaqui, and cará-trovão (*Astronotus ocellatus*) began to appear in the river, apparently as a result of fish stocking of local reservoirs in the proximity of the Capibaribe River by the government, in 1994. Up until 1998, the river was still considered to be clean and well-stocked with fish, but in 1998 and 1999, major growth in the local population resulted in an increase of the discharge of sewage, garbage, and dead animals into the river (Table 3). This period was recognized universally as the moment of transition in the quality of the river.

This increase in the amount of waste and effluents discharged into the river continued into 2000, as the population continued to grow (Table 3). From this time onward, the river was polluted, presenting the same characteristics as in the present day. In 2000, the informants reported that a regular refuse collection service began in the community, resulting in a reduction in the amount of garbage ejected directly into the river. In 2004, a species of fish known locally as Beta, appeared in the river. The presence of this fish was associated with sewage outlets.

**Fig. 6:**
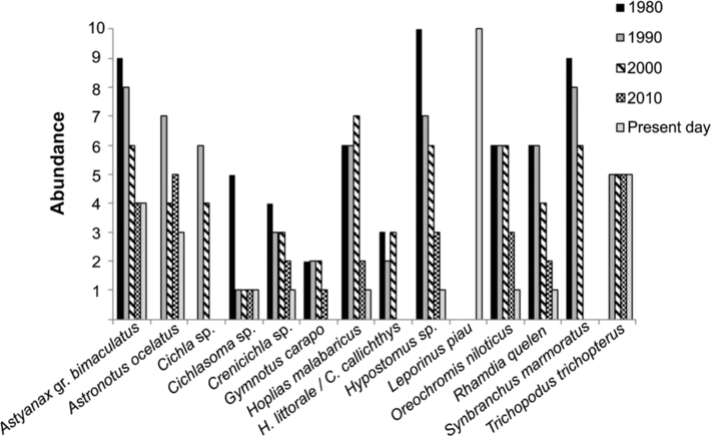
“Historical Graph” of the occurrence of the fish species confirmed in the Checklist. “Historical Graph” of the occurrence of the fish species confirmed in the Checklist. Beira Rio community resident’s yard, in lower Capibaribe River in the municipality of São Lourenço da Mata, Pernambuco, Brazil. Photographed in 2014. Beira Rio community resident’s yard, in lower Capibaribe River in the municipality of São Lourenço da Mata, Pernambuco, Brazil. Photographed in 2014

A total of 18 fish species were listed, of which 15 were confirmed by the checklist (Table 4). These 15 species were used to construct a Historical Graph (Fig. 6), which showed that, in most cases, the abundance of the species was modified over the course of the years. The Carpa and Beta species mentioned in discourse collective not were inserted in these analysis because they were not cited in freelists. The abundance of 13 of these species declined gradually over time, while that of *Trichopodus trichopterus* remained constant since its appearance in the area (Fig. 6). *Cichla* sp. and *Astronotus ocellatus* also appeared in the study area in the 1990s, coinciding with the government’s restocking program. *Cichla* sp., *Hoplosternum littorale*, *Callichthys callichtys*, *Synbranchus marmoratus*, and *Gymnotus* sp. were not reported in the present day, which may indicate their disappearance from the study area, while *Leporinus piau* was only reported after 2010, impeding any analysis of changes in its abundance.

**Table 4:** Fish species identified in the free list

Local name	Species	Family
Acari^a^	*Hypostomus* spp*.*	Loricariidae
Camurim	*-*	-
Cará	*-*	-
Cará-branca/Tilápia^a^	*Oreochromis* sp*.*	Cichlidae
Cará-preta^a^	*Cichlasoma* sp*.*	Cichlidae
Cará-trovão^a^	*Astronotus ocellatus*	Cichlidae
Cascudo^a^	*Hoplosternum littorale/Callichthys callichthys*	Callichthyidae
Guaru	*-*	-
Jacunda^a^	*Crenicichla* sp*.*	Cichlidae
Jundiá^a^	*Rhamdia quelen*	Heptapteridae
Mussum^a^	*Synbranchus marmoratus*	Synbranchidae
Piaba^a^	*Astyanax gr. bimaculatus*	Characidae
Piau/Pintado^a^	*Leporinus piau*	Anostomidae
Sarapó^a^	*Gymnotus* sp.	Gymnotidae
Tacunaré^a^	*Cichla* sp.	Cichlidae
Tambaqui	*-*	-
Traíra^a^	*Hoplias malabaricus*	Erythrinidae
Tricongati^a^	*Trichopodus trichopterus*	Osphronemidae

Fish species identified in the free list. ^a^Confirmed by the Checklist

The most abundant fish species present in the study area when the community was established were *S. marmoratus*, *Hypostomus* spp., and *Astyanax* gr. *bimaculatus*, whereas nowadays, *L. piau* is the most abundant. The predominance of *L. piau* is related to its adaptability, given that it “grows rapidly”, “produces lots of eggs”, “breeds a lot”, and “eats the eggs and young of other fishes”. According to the informants, *L. piau* arrived in this stretch of the river only two or three years ago, following the last major flood that occurred in the region, in 2011.

## Discussion

Urbanization is a multidimensional global process linked to the ongoing growth in human population and alterations in land use, a rapid and dynamics process, which may be difficult to predict [29]. The urban growth in the studied site was evident through the GIS analysis and the local perception. However, despite the complementarity and concordance of these data, the urban expansion of community Beira Rio that occurred in the late twentieth century was most evident from local perception. This local perception was possibly influenced by urban growth that also occurred in community surroundings, since according to Fernandes et al. [30] one’s perception is based on the entire visual environment surrounding him or her.

Urban expansion results in an increase in the production of waste, which is often disposed directly onto the ground or into bodies of water [31], as observed in the present study, given the lack of basic public sanitation in the community, with effluents being discharged directly into the Capibaribe. The highest levels of fecal coliforms recorded in the present study corresponds to 160 times the maximum value permitted under Brazilian federal legislation [22], reflecting the enormous quantities of domestic effluents being discharged into the river. Due to the synergistic and cumulative effects of river pollution (eg. [32, 33]) these high contamination levels recorded possibly resulted from the discharge of domestic effluents by several urban centers upstream of the Beira Rio community. However, CPRH sampling station is located next to the Beira Rio community, so the recorded levels of fecal contamination possibly have higher contribution from waste dumped by the community.

The lack of basic sanitation and the inadequate disposal of urban waste are recurring problems in developing countries, where the scarcity of financial and technical resources limits the potential for the satisfactory processing and disposal of the residues produced by the urban population [34]. The peripheral and unplanned growth of most Brazilian cities, together with the lack of basic infrastructure, have contributed to the present scenario, in which only half of the country’s municipalities has public sanitation systems, and even where they do exist, most of the effluents receive inadequate treatment before being discharged into bodies of water [21]. According to local informants in the present study, there is a constant risk of schistosomiasis from contact with the river water, as confirmed by the high rates of contamination documented in the records available for the town of São Lourenço da Mata [35]. The establishment of endemic diseases, such as schistosomiasis, is related to a complex of biological, social, political, and cultural factors, which are expressed in the living conditions of the local population [36]. This reinforces the need for basic sanitation, not only for the recuperation of the environment, but also to guarantee public health.

Any ecosystem will have a natural capacity of resisting to degradation, stabilizing or assimilating the substances found in urban effluents, and maintaining the quality of the environment [37], although the capacity for depuration of aquatic ecosystems may soon fall behind the increasing demands of urban development [38]. In the present case, while the informants referred to the degradation of the river only by the end of the 1990s, it seems likely that the gradual accumulation of waste over the preceding years eventually exceeded the depuration capacity of the river by the late 1990s. According to the available data, fecal contamination of the river already exceeded legal limits at the beginning of the 1990s, although a peak in values was recorded between 1996 and 2000, corresponding approximately to the period of greatest environmental disturbance indicated by the informants. In addition, the river is suffering an ongoing process of eutrophization which, according to CETESB [39] may have a series of negative consequences for the river and its resources, including increased fish mortality, an increase in algal biomass, and blooms of cyanobacteria. The eutrophization of bodies of water is a recurring problem in urbanized environments, especially densely-populated areas with an intense discharge of nutrients, as observed in China by Gao and Zhang [40].

The loss of water quality affects the supply of ecosystem services to human populations, given that these services depend on the physical,chemical and biological conditions of the ecosystem [9]. The bacteriological conditions of the Capibaribe recorded in the present study are consistent with the prohibition of all activities except navigation and landscape harmony, according to Brazilian legislation [22].

The emphasis on ecosystem services reinforces the understanding of the relationship between the local population and the environment, in terms of its dependence on natural resources [41]. The present study demonstrated that the degradation of the aquatic environment resulted in a loss of environmental services, modifying the habits of the local population, and their feelings in relation to the river. During the early years of the community, the residents had positive feelings toward the river, which they used for domestic supplies, drinking water, fishing, and recreational activities, whereas at the present time, the river has a negative image, being considered unpleasant and useless. The negative modifications of the river are related primarily to the color and odor of the water, as observed by Johnson et al. [42] in an urban river basin, where the changes in the quality of the aquatic ecosystem were perceived by local residents primarily through their visual or olfactory perceptions. Additional changes perceived by the local population in this study were in the sediment and fish population. The accumulation of fine sediments and alterations of the natural riverbed are recurring problems in urbanized systems [43].

The pollution of bodies of water may render fishing unviable due to the loss of species [8] and the reduction in the quality of fishes [44]. These factors were cited among the reasons for abandoning of fishery activities in the study area. The loss of fish species is a common problem in degraded environments [45]. Despite the importance of the Capibaribe River to the state of Pernambuco, there are no previous studies that provide information on the diversity of its ichthyofauna, being the results of this research the only source available to understand the local fish fauna.

Fish are no longer considered to be a source of food by the informants from the Beira Rio community, due to the contamination of the water. A number of studies have shown that the fishes can become contaminated by the discharge of pollutants into aquatic ecosystems [46]. Given the potential risks to human health and wellbeing, many populations that once depended primarily on fishery resources for their diet have abandoned this practice altogether [47].

Exotic species may be introduced by man either intentionally or accidentally [48]. The restocking of freshwater systems has been promoted by the Brazilian government as a strategy to guarantee the survival of artisanal fishermen by providing a source of income and employment [49]. During the 1990s, thousands of fingerlings were released into ponds and reservoirs in Pernambuco, resulting in the dispersal of species such as curimatã (*Prochilodus* sp.), tambaqui (*Colossoma macropomum*), tilapia (*Oreochromis* sp.), carp (*Cyprinus carpio, Hypophthalmichtys molitrix* or *Aristichthys nobilis*), and piau (*Leporinus* sp.) [49]. Some of these species were reported being found in the Beira Rio community during this same decade, except for the piau (*L. piau*), which appeared only two or three years ago, following a flood. The arrival of *L. piau* may have resulted from the accidental connection of the main channel of the river to fish farms or reservoirs located within the area flooded by the river. Fish farming is a common practice in many parts of Brazil, although the government agencies responsible for the monitoring of this activity do not control the dispersal of species, and lack environmental management requirements [50].

Exotic fish species may cause ecological damage in new habitats, generally through an increase in competitive and predation pressures [51]. *Leporinus piau* represents a threat to the other fish species due to its generalist habits and voracious appetite [52], which enhance its potential competitiveness in comparison with the resident species. Despite this situation, informants considered positively the presence of *L. piau*, due to the quality of its flesh.

## Conclusions

The degradation of an urban river had negative effects on the river and its ichthyofauna, affecting the subsistence and intimate relationship of the adjacent human community with this environment. Despite the current degraded condition of the river, the local community still harbors hope that this river will someday become a pleasant environment for the local community, as long as the factors causing the degradation of the environment are corrected, in particular by building a public sanitation system, which would reduce the discharge of domestic effluents directly into the river. In general, however, no real expectation was found of a return to a fishery-based economy, especially as the locals have already adopted other means of subsistence. The current situation on this river emphasizes the urgent need for the establishment of mitigating measures in order to avoid its complete degradation. These measures will require efforts on social, cultural, and political fronts for the development of programs that contribute to the improvement of the quality of both the environment and the lives of local residents. In particular, there is a clear need for public works of infrastructure, in particular a sanitation system, combined with efforts to modify the consciousness of the community with regard to the need to avoid the discharge of waste into the river, and encourage the participation of local residents in recuperation and conservation projects. The integrated approach used in this study demonstrated the complementarity of the obtained data, which provided an understanding of the degradation process of an urban river, revealing the effectiveness of implementation of this approach.
